# Anesthetic Considerations in Hepatectomies under Hepatic Vascular Control

**DOI:** 10.1155/2012/720754

**Published:** 2012-05-28

**Authors:** Aliki Tympa, Kassiani Theodoraki, Athanassia Tsaroucha, Nikolaos Arkadopoulos, Ioannis Vassiliou, Vassilios Smyrniotis

**Affiliations:** ^1^First Department of Anesthesiology, School of Medicine, University of Athens, Aretaieion Hospital, 76 Vassilisis Sofias Avenue, 11528 Athens, Greece; ^2^Fourth Department of Surgery, School of Medicine, University of Athens, Attikon Hospital, 1 Rimini Street, 12410 Chaidari, Greece; ^3^Second Department of Surgery, School of Medicine, University of Athens, Aretaieion Hospital, 76 Vassilisis Sofias Avenue, 11528 Athens, Greece

## Abstract

*Background*. Hazards of liver surgery have been attenuated by the evolution in methods of hepatic vascular control and the anesthetic management. In this paper, the anesthetic considerations during hepatic vascular occlusion techniques were reviewed. *Methods*. A Medline literature search using the terms “anesthetic,” “anesthesia,” “liver,” “hepatectomy,” “inflow,” “outflow occlusion,” “Pringle,” “hemodynamic,” “air embolism,” “blood loss,” “transfusion,” “ischemia-reperfusion,” “preconditioning,” was performed. *Results*. Task-orientated anesthetic management, according to the performed method of hepatic vascular occlusion, ameliorates the surgical outcome and improves the morbidity and mortality rates, following liver surgery. *Conclusions*. Hepatic vascular occlusion techniques share common anesthetic considerations in terms of preoperative assessment, monitoring, induction, and maintenance of anesthesia. On the other hand, the hemodynamic management, the prevention of vascular air embolism, blood transfusion, and liver injury are plausible when the anesthetic plan is scheduled according to the method of hepatic vascular occlusion performed.

## 1. Introduction

Hepatectomy is one of the therapies available for benign and malignant liver disease. Although liver resections have been associated with high mortality and morbidity rates, recent advances in anesthetic and surgical management have significantly reduced the operative risk. The techniques of vascular control during hepatectomy are highly demanding and should be performed under special anesthetic considerations.

 Hepatic vascular control methods can be categorized as those involving occlusion of liver inflow and those involving occlusion of both liver inflow and outflow. They can be summarized as following.

1. Inflow vascular occlusion.
  - Hepatic pedicle occlusion:
    - Continuous Pringle maneuver (CPM),
    - intermittent Pringle maneuver (IPM).
  - Selective inflow occlusion.
2. Inflow and outflow vascular exclusion
  - Total hepatic vascular exclusion (THVE),
  - inflow occlusion with extraparenchymal control of the major hepatic veins: with selective hepatic vascular exclusion (SHVE).

When performing these techniques, the conduct of anesthesia should take into account hemodynamic management, risks of vascular air embolism, ischemia reperfusion liver injury, intraoperative blood loss, and the need for transfusion, factors which usually complicate hepatic vascular control methods. Special attention should also be paid to the preoperative assessment and induction of anesthesia, as patients undergoing liver resection usually have a compromised health status. Careful selection of the anesthetic drugs can minimize the effects of hepatic blood flow decrease induced by the surgical technique adopted.

## 2. Methods

 A comprehensive literature search was performed. Our objective was to identify the anesthetic considerations in techniques of hepatic vascular control methods. Articles were selected by a Medline literature search, according to the following criteria.

1. All prospective randomized studies were thoroughly evaluated and presented, as they are the most important source of information on the outcomes of surgical and anesthetic manipulations.
2. Large retrospective studies were also included. Few case reports and smaller studies are mentioned, given the fact that they highlight special anesthetic aspects.

## 3. Results

### 3.1. Preoperative Assessment

 Healthy patients undergo a routine preoperative assessment including a full blood count and a standard biochemical and coagulation test.

 Preexisting hepatic impairment is a risk factor, even for nonhepatic surgery, with higher blood transfusion requirements, a longer hospital stay, a higher number of complications, and increased mortality rates of 16.3% in cirrhotic patients as compared to 3.5% in controls [1]. Estimating the health status of patients presenting for hepatectomy is quite challenging: coagulopathy, volume and electrolyte disturbances, viral infections (Hep C), hepatorenal [2–4] and hepatopulmonary [3] syndrome, portopulmonary hypertension, and low cardiovascular reserve capacity can occur in patients with chronic liver disease.

The identification of patients at risk to develop postoperative hepatic or renal failure is important and, ideally, involves many related disciplines such as surgery, anesthesia, and intensive care. Although vascular occlusion techniques have minimized hepatic bleeding, the risk for postoperative liver and/or renal failure remains high for patients of advanced age and those with steatosis and cirrhosis, on preoperative chemotherapy and with small remnant liver volumes [5]. Slankamenac et al. [6] have developed and validated a prediction score for postoperative acute liver failure following liver resection based on the preoperative parameters of cardiovascular disease, chronic liver failure, diabetes, and ALT levels, which seems to be an easily applicable and attractive tool in clinical practice.

 Vascular control techniques during hepatectomy require optimization of the cardiac and pulmonary function [7]. Hepatic ischemia and reperfusion on subsequent liver dysfunction is associated with unexpected responses to surgical stress [7–9] and poor prognosis [10]. Patients with end-stage liver disease have a characteristic hemodynamic profile: increased cardiac output with blunted response to painful stimuli, splanchnic vasodilatation and central hypovolemia. As a result, silent moderate-to-severe coronary artery disease cannot be easily recognized. Currently, there are no specific guidelines for the identification of coronary artery disease in patients with advanced liver disease [11, 12]. Preoperative invasive assessment of preexisting cardiovascular dysfunction is indicated only for high risk patients, provided that any coagulopathy is corrected [11]. In the noninvasive assessment of coronary artery disease in patients with cirrhosis, dobutamine stress echocardiography has failed as a screening tool [12]. Furthermore, beta blockade discontinuation in order to permit adequate cardiac function assessment may be hazardous in patients with advanced liver disease [12]. Beta blockers reduce portal hypertension, decrease cardiac workload, and their use seems to be beneficial to both the liver and the heart in the setting of hepatectomy.

 In general, the preoperative assessment needs to be adapted to the individual patient to minimize the perioperative liver insults of hepatic vascular control.

### 3.2. Induction and Maintenance of Anesthesia

 Liver resections are usually performed under general anesthesia with tracheal intubation and controlled ventilation. Patients with ascites undergo rapid sequence induction [13]. Cis-atracurium is the nondepolarizing muscle relaxant of choice in patients with liver disease as it is hydrolyzed by Hoffman elimination. Moreover, it is haemodynamically stable due to its scarce release of histamine [14]. Atracurium can provide stable neuromuscular blockade, as its requirements remained unchanged during exclusion of the liver from the circulation [15].

 An intravenous hypnotic is used for induction and a halogenated volatile agent in air-oxygen mixture is used for maintenance [16]. Hepatic vascular control techniques depress cardiovascular function in addition to the depression caused by general anesthesia. Careful selection of the volatile agent is required. Most commonly used volatile anesthetics for maintenance are isoflurane and sevoflurane. Isoflurane has mild cardiodepressive effects but maintains hepatic oxygen supply, due to vasodilatation in the hepatic artery and portal vein [17]. Both isoflurane and sevoflurane upregulate heme-oxygenase-1, release iron and carbon monoxide, and thus decrease portal vascular resistance in rats [18]. In humans, sevoflurane decreases portal vein blood flow but increases hepatic artery blood flow [19]. In addition, Beck-Schimmer et al., in a randomized controlled trial on patients undergoing liver surgery [20], showed that ischemic preconditioning with sevoflurane before inflow occlusion limited postoperative liver injury, even in patients with steatosis. Although various inhalational anesthetics are used in liver surgery, no optimal anesthetic technique has been established for the maintenance of anesthesia. Desflurane appears to have no greater liver toxicity than currently used volatile anesthetic agents [21]. Additionally, desflurane undergoes only minor biodegradation (it is metabolized at a ratio of 0.02%) and in fact it may cause less hepatocellular damage due to its reduced metabolism [21]. Ko et al. [22], comparing the effects of desflurane and sevoflurane on hepatic and renal functions after right hepatectomy in living donors reported better postoperative hepatic and renal function tests with desflurane as compared to sevoflurane at equivalent doses of 1 MAC without, however, being able to validate the clinical importance of their study. Arslan et al. [23] comparing the effects of anesthesia with desflurane and enflurane on liver function, showed that during anesthesia with desflurane, liver function was well preserved; glutathione-S-transferase and aspartate aminotransferase levels were significantly lower in the desflurane group. On the other hand, Laviolle et al. [24] suggested that propofol has an early protective effect against hepatic injury compared with desflurane after partial hepatectomy under inflow occlusion.

 It is now generally accepted that anesthesia reduces hepatic blood flow. However, few studies on the effects of general anesthesia during hepatectomies under vascular control techniques are available in patients with significant comorbidities.

### 3.3. Hemodynamic Management

#### 3.3.1. Inflow Vascular Occlusion

CPM, IPM, and selective inflow occlusion share common hemodynamic management. Portal triad clamping increases systematic vascular resistance by up to 40% and reduces cardiac output by 10%. Mean arterial pressure increases about 15% (Table 1). Following unclamping, hemodynamic parameters gradually return to baseline values [25–28]. However, the systemic circulation in patients with cirrhosis is hyperdynamic and dysfunctional, with increased heart rate and cardiac output, decreased systemic vascular resistance, and low or normal arterial blood pressure. Thus, maintaining adequate organ perfusion may be difficult to achieve and preoperative optimization of the patient is required.

 The anesthetic management is dictated by the surgical approach and the patient's health status. For healthy patients, routine monitoring is used. Monitoring can even be limited to just peripheral vein catheters [29]. Invasive monitoring provided by a central venous line or pulmonary catheterization is reserved for patients with poor cardiovascular status or when prolonged vascular occlusions are performed.

A low CVP (between 2 and 5 mmHg), while aiming at euvolemia, reduces blood loss during liver surgery and improves survival [30, 31]. A low CVP can be achieved by limitation of intravenous fluids administration pre- and intraoperatively. Maintenance fluids and crystalloids to stabilize blood pressure >90 mmHg and ensure diuresis of at least 0.5 mL/kg/h can be used safely with minor hemodynamic disturbance [32]. If fluid restriction is ineffective to keep a low CVP, vasoactive agents are used. Nitroglycerin reduces CVP to the desired level during the resection phase or when excessive oozing is observed from the resected surface [13, 16]. Intravenous morphine has also been used for its hypotensive effect.

 CPM with a CVP of 5 mmHg or less is associated with minor blood loss and a shorter hospital stay [33]. IPM may result in fluctuations of systemic blood pressure. If, however, it is applied under a low CVP during transection, blood loss and hemodynamic changes are minimal [34–37]. In an experimental animal study, Sivelestat, a neutrophil elastase inhibitor, reduced hepatic injury and stabilized hemodynamics after ischemia-reperfusion following IPM [38].

 The advantages of a low CVP must be weighed against inadequate perfusion of the vital organs and loss of volemic reserve in case of bleeding and/or air embolism. A 15° Trendelenburg position protects against air embolism. Melendez et al. [34] support that in low CVP anesthesia during liver resection, the incidence of perioperative renal failure does not increase significantly.

#### 3.3.2. Inflow and Outflow Vascular Occlusion

**(1) Total Hepatic Vascular Exclusion (THVE)**

In THVE, rapid hemodynamic changes (Table 1) are frequent due to surgical events such as caval clamping, sudden blood loss, and hepatic reperfusion. Cross-clamping of the inferior vena cava and portal vein result in a 40–60% reduction of venous return and cardiac output, with a compensatory 80% increase in systemic vascular resistance and a 50% increase in heart rate. Although systemic vascular resistance and heart rate increase, the cardiac index is reduced by half, secondary to a preload reduction. Unclamping is followed by an increase in cardiac index and a significant reduction in systemic vascular resistance [39].

The anesthetist should take prompt steps to manage the preload reduction and the sudden decrease in cardiac output evoked by the inferior vena cava and portal vein clamping. Intraoperative monitoring includes ECG, pulse oximetry, ETCO_2_ tension, invasive blood pressure monitoring through an arterial line, and CVP monitoring through a large bore central venous line. Patients with pulmonary hypertension require pulmonary artery catheterization. In addition, the presence of a pulmonary artery catheter allows the tailored administration of vasopressors in case of massive hemorrhage due to vena cava injury. The Vigileo, an uncalibrated arterial pulse contour cardiac output monitoring system, has been proved to be unreliable in cirrhotic patients with hyperdynamic circulation undergoing major liver surgery [40].

Before THVE, colloids can be administered to prevent the abrupt decrease in cardiac output. Colloids, beyond correcting volume deficits [33], improve splanchnic circulation, displace fluid into the blood compartment, and reduce bowel edema. Blood pressure and circulatory support is achieved by aiming at a CVP of at least 14 mmHg [16]. Vasopressin or norepinephrine are administered if volume loading is inadequate to maintain blood pressure following clamping of the vena cava [7].

There is no standard approach to the use of vasoactive agents in THVE. Most studies have mainly been performed in septic patients or in animal models. Vasoactive agents should be used carefully, as they improve cardiac output at the expense of microcirculatory blood flow. During vascular isolation of the liver in eight pigs, norepinephrine infusion (0.7 *μ*g/kg/min) decreased hepatic vascular capacitance by activation [41]. In a recent study in septic patients, Krejci et al. [42] showed that norepinephrine increased systemic blood flow but reduced microcirculatory blood flow on liver's surface.

Vasopressin on the other hand, is known to rapidly restore blood pressure during septic shock. However, in an experimental study [43], vasopressin proved to be inferior to norepinephrine in terms of improving hepatosplanchnic blood flow. The response to both norepinephrine and vasopressin is blunted in patients with cirrhosis [44, 45].

Preventing renal impairment is another important consideration for the anesthesiologist. Renal autoregulation ceases below a renal perfusion pressure of 70 to 75 mmHg, below which, flow becomes pressure dependent. Perioperative fluid shifts, intravascular hypovolemia, and sympathetic activation during THVE result in a reduction of renal blood flow. Mannitol, furosemide, and “low dose dopamine” have been used with the aim of preventing intraoperative renal injury without evidence of substantial benefit [46]. Fenoldopam had beneficial effects [47] on postoperative creatinine levels and creatinine clearance of critically ill patients [48]. Recently, terlipressin along with volume expansion have been shown to improve renal function, without, however, improving survival [49].

Hemodynamic intolerance to THVE or ischemia under THVE exceeding 30 or 60 minutes, require venovenous bypass [50, 51]. THVE should be limited to selected cases, as hemodynamic intolerance has been observed in 10–20% of patients, as well as increased morbidity and hospital stays (Table 2).

**(2) Selective Hepatic Vascular Exclusion (SHVE)**

SHVE is a flexible technique that can be applied in a continuous or intermittent manner. Should accidental tears of major hepatic veins occur, rapid conversion to THVE must be undertaken. The literature suggests that many institutions favor SHVE as one of the standard methods of vascular control because it provides a bloodless surgical field and it is tolerated by most patients. No special anesthetic considerations regarding the hemodynamic management of SHVE are referred, as this method diminishes blood pressure and heart rate fluctuations during liver resection (Table 1).

In a cohort study [52] among 246 patients, hemodynamic tolerance to SHVE was excellent with only a slight increase in systemic and pulmonary resistance during clamping. No deaths were reported and the mean hospital stay was 9.6 days.

SHVE is the method of choice in cases when CVP cannot be lowered (i.e., right heart failure, poor cardiovascular status) [53–56]. In a retrospective study on 102 patients, SHVE was shown to be unaffected by CVP levels and the authors concluded that it should be used whenever CVP remains high despite adequate anesthetic management [57]. Although the performance of SHVE requires significant surgical expertise, it is tolerated by most patients and has a hemodynamic profile similar to that of CPM [53, 54]. Furthermore, it controls backflow bleeding of the hepatic veins. In a large clinical study [58], SHVE proved to be more effective than CPM in controlling intraoperative bleeding, preventing blood loss, and reducing postoperative complications and mortality rates (Table 2). Combined SHVE and perioperative fluid restriction has also been suggested as a liver and renal protective procedure in partial hepatectomy. Moug et al. [59] demonstrated that active preoperative dehydration of the patient, low CVP anesthesia and SHVE resulted in minimal blood loss, low morbidity, and zero mortality in patients undergoing partial liver resection.

In conclusion, SHVE which is not associated with cardiorespiratory and hemodynamic alterations is well tolerated by the majority of patients and requires shorter hospitalization times [54].

### 3.4. Vascular Air Embolism

 Although the relative risk of air embolism in hepatic surgery is low (<5%) [60], several cases have been reported during liver vascular control techniques. Factors predisposing to vascular air embolism during liver resections include: (a) surgical technique, (b) size and place of the tumor, (c) blood loss, and (d) low CVP anesthesia.

Clinical signs of vascular air embolism during anesthesia with respiratory monitoring are: a decrease in end-tidal carbon dioxide and decreases in both arterial oxygen saturation (SaO_2_) and tension (PO_2_), along with hypercapnia. From the cardiovascular system monitoring, tachyarrhythmias, electromechanical dissociation, pulseless electrical activity as well as ST-T changes can be noted. Major hemodynamic manifestations such as sudden hypotension may occur before hypoxemia becomes present.

 When performing techniques of inflow vascular occlusion (CPM, IPM, selective inflow occlusion), air embolism may be observed during parenchymal transection under low CVP anesthesia or during reperfusion, due to mobilization of air bubbles trapped in opened veins. Resection of large tumors situated in the right lobe [61], close to the inferior vena cava or the cavohepatic junction, put the patient at risk of venous air embolism. Those tumors should therefore be resected under THVE or SHVE if possible. Recent clinical trials assessing the efficacy of SHVE and Pringle maneuver in preventing vascular air embolism showed that embolism occurred in three out of 2100 patients or in one out of 29 patients of the Pringle group, following massive blood loss during tumor resection. Air embolism did not occur in any case of the SHVE group [62–64]. 

 Massive bleeding (>5000 mL) and subsequent air embolism can even result in intraoperative death in patients undergoing major liver resections [65]. The morbidity and mortality of air embolism depend on the volume and rate of air accumulation [66]. From case reports of accidental intravascular delivery of air, the adult lethal volume has been described as between 200 and 300 mL or 3–5 mL/kg [67, 68]. Low CVP further enhances the negative pressure gradient at the surgical field compared to the right atrium and increases the possibility of air embolism.

 Currently, the most sensitive monitoring devices for vascular air embolism are transesophageal echocardiography and precordial Doppler ultrasonography, detecting as little as 0.02 mL/kg and 0.05 mL/kg of air, respectively [69–71].

 The consequences of air embolism can be minimized by placing the patient in a 15 degree Trendelenburg position [72–74]. However, recent literature has questioned the efficacy of Trendelenburg position on improving hemodynamics [75]. Furthermore, Moulton et al. [75] in a small study among ten patients, showed that patient positioning alone during liver surgery does not affect the risk of venous air embolism. Thus, the beneficial effects of low CVP in liver resections must be carefully weighed against adequate hydration and volume status optimization.

 Vascular air embolism is a potentially hazardous complication. Additionally, cirrhotic patients undergoing hepatectomy have pulmonary abnormalities including intrapulmonary shunting, pulmonary vascular dilatation, and arteriovenous communications. In these patients, air can pass into the systemic circulation (paradoxical air embolism), even if cardiac abnormalities (patent foramen ovale) are not present, evoking fatal consequences [76].

 Recent literature suggests that SHVE prevents vascular air embolism and provides operative tolerance. However, recognizing the risk for vascular air embolism and planning the appropriate level of monitoring and treatment is the key to patient safety.

### 3.5. Blood Loss and Transfusion

 Liver resections may result in significant blood loss and subsequent transfusion of RBC (red blood cells) in about 25%–30% of patients [77]. The two main sources of bleeding during a liver resection are (a) the inflow system (hepatic artery and portal vein) and (b) the outflow system (backflow bleeding from the hepatic veins). Bleeding may also occur during liver mobilization, hepatic transection, and dissection of biliary structures.

 Blood loss has been linked to morbidity and mortality since 1989 [8], whereas RBC transfusions are associated with multiple disadvantages, risks, and side effects. Furthermore, operative blood loss independently predicts recurrence and survival after resection of hepatocellular carcinomas [78]. Operative mortality in patients refusing blood transfusions was 7.1% for patients with hemoglobin levels >10 g/dL and 61.5% for patients with hemoglobin levels <6 g/dL [79, 80].

 The refinement of inflow and outflow occlusive techniques as well as the appropriate anesthetic management has reduced intraoperative bleeding and the need for blood transfusions. The surgical approach to hepatic resection is of major importance in preventing blood loss. Study of the literature reveals the following results regarding bleeding with different vascular occlusion techniques: Pringle maneuver has been shown to be effective in reducing blood loss during parenchyma transection [81]. Portal triad clamping is associated with less bleeding compared with no clamping [82]. In procedures of liver ischemia time < one hour, CPM is equal to IPM. Belghiti et al. [9], in a prospective study of IPM versus CPM, found no difference in total blood loss or the volume of blood transfused between the two groups, despite higher blood loss during parenchyma transection. Man et al., in two prospective studies of IPM versus no use of vascular control at all, showed lower total blood loss and fewer transfusions in the IPM group [83–85]. Hemihepatic vascular clamping was shown superior to IPM and to no application of vascular control, with reduced both blood loss and transfusion requirements [86]. SHVE provides a bloodless surgical field similar to THVE, but is better tolerated by patients. Many authors favor SHVE as one of the standard methods of vascular control, as it substantially prevents massive blood loss and diminishes transfusion needs.

 From an anesthetic standpoint, a low CVP level plays an important role in reducing intraoperative blood loss and transfusion rates [30, 57, 87]. Maintaining a CVP < 5 mmHg by volume restriction and intravenous infusion of nitroglycerine and a systolic blood pressure above 90 mmHg by intravenous infusion of dopamine (4–6 *μ*g/kg) has dramatically reduced bleeding and transfusion requirements [88]. The anesthetist should also provide normothermic conditions to the patient undergoing liver resection, because hypothermia reduces blood coagulation, especially platelet function, and increases intraoperative blood loss.

 Alternative methods of diminishing blood loss have been investigated. Of the pharmacological methods, desmopressin, although used in treating hemophilia, was not effective in reducing blood loss and transfusion needs in patients undergoing liver resection. In a randomized clinical trial, the use of recombinant factor VIIa in major liver resections failed to reduce the number of units transfused [89]. A significant reduction in blood transfusion needs in liver resections has been shown with the use of aprotinin. Aprotinin was found to reduce intraoperative blood loss by 25% and transfusion requirements by 50% [81]. Redai et al. [16] used half dose aprotinin (10^6^ KIU followed by 2.5 × 10^5^ KIU/hour infusion) during hepatic transplantation in patients who have a significant coagulopathy or portal hypertension and in those who had previous abdominal surgery. However, Lentschner et al. [90] cautioned against the routine use of aprotinin due to the incidence of life threatening allergic reactions, thrombotic potential, and renal failure. Currently, there is no scientific support for the routine use of aprotinin in patients undergoing partial hepatectomy, whereas its efficacy in liver transplantation is well established [91]. Tranexamic acid has also been shown to reduce blood requirements in liver resection surgery but safety concerns have been raised and require further investigation [92, 93]. In the future, two artificial oxygen carriers (hemoglobin solutions and perfluorocarbons) may become essential in reducing the need for allogeneic RBC transfusions [94–96]. Artificial oxygen carriers improve O_2_ delivery and tissue oxygenation as well as the function of organs with marginal O_2_ supply. More studies examining their efficacy in ischemic liver during hepatectomy need to be performed.

 Undoubtedly, the improvement of vascular control techniques during hepatectomy has permitted an aggressive approach for liver resections with low mortality rates (4%) [52]. In addition, anesthesia orientated towards an almost transfusion free setting has also improved mortality and morbidity following liver surgery. To this direction, Pulitanò et al. [97] proposed a score predicting blood requirements in liver surgery. A transfusion risk score, including variables of: (a) preoperative hemoglobin concentrations below 12.5 g/dL, (b) largest tumor more than 4 cm, (c) need for exposure of the vena cava, (d) need for an associate procedure, and (e) cirrhosis, accurately predicted the likelihood of blood transfusions in liver resections.

 Recently, Cescon et al. [52], in a retrospective review assessing the outcome of 1500 consecutive patients who underwent hepatic resection, estimated overall mortality and morbidity at 3% and 22.5%, respectively. Their multivariate analysis revealed that blood transfusions, primary liver tumors, and additional procedures were associated with an increased risk of postoperative complications, whereas blood transfusions, cirrhosis, biliary malignancies, and extended hepatectomy were associated with an increased risk of postoperative mortality. Wang et al. [98], evaluating the long-term outcomes of liver resection for hepatocellular carcinoma, estimated that 86.9% of the patients did not require perioperative blood transfusion and that Pringle maneuver and RBC transfusions are independent prognostic factors influencing survival.

 Blood transfusions are well known to carry the risk of transmitted infections, acute or delayed reactions and “wrong blood” incidents. In liver resections, blood transfusions are associated with suppression of the immune system. There is strong evidence that blood transfusions have an impact on tumor recurrence for patients with early stages of hepatocellular carcinoma. However, no such effect could be demonstrated for patients undergoing partial liver resection for late stages of hepatocellular carcinoma, colorectal metastasis, or cholangiocarcinoma [99]. Transfusion evoked immunosuppression is also responsible for TRALI (transfusion-related acute lung injury). Dyspnea, hypotension, fever, and bilateral noncardiogenic pulmonary edema, present within 6 h of transfusion and complicate the postoperative outcome of patients following major liver surgery [100]. Patients with chronic liver disease have the greatest risk of developing TRALI, in comparison to other populations [101, 102]. Although all blood products can lead to this life-threatening situation, plasma-containing products were responsible for the majority of cases in patients undergoing liver transplantation [101]. Recent studies suggest that TRALI fatalities followed plasma transfusion components were linked to multiparous female donors with leukocyte antibodies [103, 104]. Therefore, the establishment of new strategies in blood donation excluding multiparous women as donors, as potential carriers of TRALI-inducing antibodies, is expected to eliminate this entity.

 In conclusion, given the influence of blood loss and transfusions on the surgical outcome, techniques of liver vascular control and anesthetic management should be adjusted to the individual patient. The tumor location, the underlying liver disease and the patient's cardiovascular status should therefore be taken into account, in order to minimize blood loss and transfusion requirements.

### 3.6. Ischemia-Reperfusion Injury and Preconditioning

Ischemia/reperfusion (I/R) injury is a serious complication of liver surgery, especially after extended hepatectomies [105]. It causes a local and systemic inflammation response and its clinical manifestations may vary from transient arrhythmias to multiorgan dysfunction and death [106]. Reperfusion injury is mediated via reactive oxygen species which damage cellular membranes, stimulate leukocyte activation and endothelial adhesion, and activate the complement. All these pathophysiological changes lead to microcirculatory failure. Hepatic I/R injury affects patient recovery after major surgery and bears a risk of poor postoperative outcome [107]. In liver surgery, ischemic preconditioning (IP) and intermittent clamping are the only established methods to provide protection against tissue damage due to ischemia during inflow occlusion [98, 108].

 IP is defined as a process in which a short period of ischemia, separated by intermittent reperfusion, renders an organ more tolerant to subsequent episodes of ischemia [107, 109]. It was initially described for a canine heart by Murry et al. in 1986 [110]. As far as the liver is concerned, the beneficial effect of IP was first demonstrated in a rodent model by Lloris-Carsi et al. [111]. Clavien et al. provided the first clinical evidence of benefit in patients undergoing hemihepatectomy [112]. It leads to improvement of hepatic microcirculation, reduction in tissue apoptosis, and improvement of survival. Experimental data suggest that generation of adenosine, activation of adenosine A_2_ receptors with subsequent generation of NO and release of NO cause vasodilation and prevent the increase in endothelins, thus protecting the liver from reperfusion injury [107]. IP stimulates adenosine receptors on Kupffer cells in nonischemic lobes to produce oxygen radicals, leading to the promotion of liver regeneration after partial hepatectomy [113]. In a clinical study of 61 patients undergoing liver surgery performed by Heizmann et al., the absence of preconditioning was found to be an independent risk factor for postoperative complications [114]. The benefit of ischemia is restricted by old liver [109]. It has been stated that IP might also be less beneficial during extended liver resections, due to hyperperfusion-induced derangement in hepatic microcirculation. Similarly, the effect of preconditioning was lost in patients undergoing tissue loss above 50% [115]. In small liver remnants of about 30%, it may in fact have detrimental effects. This is because the small remaining tissue suffers from shear stress-associated microvascular injury. Ischemic preconditioning seems to attenuate the apoptotic response of hepatic cells in major hepatectomies performed under SHVE [115]. On the other hand, Azoulay et al. found that IP failed to protect human liver against IR injury after major hepatectomy under continuous vascular occlusion with preservation of caval flow [116]. Other strategies should be used to induce protection in this setting. Combined IP and salvialonic acid-B have been shown to possess synergistically protective effects in rats, mediated through reduction of postischemic oxidative stress, higher ATP levels and reduction in hepatocellular apoptosis [105].

 The severity of IR injury is related to the duration of vascular occlusion. The preconditioning effect fades away when the ischemic time is prolonged [108]. In this case, intermittent vascular occlusion, although more complex surgically, seems to be the method of choice. Van Wagensveld et al. demonstrated that prolonged intermittent vascular inflow occlusion in pig liver surgery caused less microcirculation impairment and hepatocellular necrosis compared with continuous occlusion and recommend it when a prolonged period of vascular inflow occlusion is expected [117]. It has been found that when ischemia persists for more than 40 minutes, intermittent vascular occlusion offers better protection of liver cells, demonstrated by lower AST values, lower apoptotic activity and reduced capsase-3 activation [108].

 In several animal models, pharmacological preconditioning with a volatile anesthetic has been proven to provide protection against ischemic injury. Beck-Schimmer et al. evaluated the effects of sevoflurane preconditioning before liver ischemia and concluded that this particular volatile anesthetic limited the postoperative increase of serum transaminase levels by 261 U/L for the ALT and by 239 U/L for the AST. The sevoflurane group had less major complications (such as sepsis, bilioma, bleeding, and infection) than the control (propofol) group. The protective effects were more pronounced in patients with liver steatosis [20]. However, according to Wang et al., propofol also seems to have the ability to protect human hepatic L02 cells from H_2_O_2_-induced apoptosis [118]. Intraportal administration of L-arginine, a precursor of NO, has been recently studied in pigs and appears to reduce cell damage during the early phase of reperfusion, by downregulating capsase-3 activty and by preserving mitochondrial structure. Clinically, it resulted in a reduction of AST and an increase in bile production [119]. In another animal study, simvastatin (5 mg/kg) protected the rat liver from I/R injury by regulating the inflammatory response and by improving microvascular flow [120]. Prostaglandins have also been found to have protective effects on I/R-injured livers by inhibiting the generation of reactive oxygen species, preventing leucocyte migration, improving hepatic insulin and lipid metabolism and regulating the production of inflammatory cytokines. They are also essential after hepatectomy because they promote hepatocyte proliferation [121].

Finally, Ramalho et al. reported that angiotensin II type I receptor (AT1R) antagonist increased regeneration in nonsteatotic livers, while in the presence of steatosis both AT1R and AT2R antagonists increased liver regeneration [122].

## 4. Conclusions

 Hepatic vascular occlusion techniques require anesthetic expertise. Intolerance to THVE is not unusual and this method should be reserved for patients in need for extensive reconstruction of the inferior vena cava. SHVE has the most favorable intraoperative and postoperative hemodynamic profile. Inflow occlusion techniques, although simple and effective, require specific anesthetic manipulations to reduce liver injury and prevent backflow bleeding.

 Every method of hepatic vascular control applied under a carefully selected anesthetic plan can improve the outcome of patients undergoing hepatectomy. The surgeon and anesthesiologist must work together effectively. Anesthetic vigilance along with thorough knowledge of the surgical manipulations promotes team-based health care in the operative room.

## Figures and Tables

**Table 1:** Hemodynamic changes on clinical series of hepatectomies induced by hepatic vascular occlusion techniques.

	Technique	Haemodynamic changes		
		Heart rate	Mean arterial blood pressure	Cardiac index
Inflow and outflow occlusion	THVE*			
	Redai et al.^a^ [16]	↑ 25%	↓ 17,64%	↓ 50%
	Smyrniotis et al.^a^ [123]	↑ 21%	↓ 23%	↓ 50%
	Figueras et al.^a^ [124]	↑ 18,75%	↓ 20,48%	↓ 60%
	Smyrniotis et al. [54]	↑ 29%	↑ 22%	↓ 50%
	SHVE**			
	Figueras et al.^a^ [124]	↑ 2,46%	↑ 3,79%	N/A
	Smyrniotis et al. [54]	↑ 5%	↑ 5,55%	↓ 10%

Inflow occlusion	Pringle			
	Redai et al.^a^ [16]	↑ 6.25%	↑ 15%	↓ 10%
	Smyrniotis et al.^a^ [123]	↑ 12%	↑ 16%	↓ 10%
	Figueras et al.^a^ [124]	↑ 8.83%	↑ 13.85%	N/A

^a^Values expressing % change of heart rate, mean arterial blood pressure, and cardiac index during clamping and uclamping of hepatic vessels.

*THVE: total hepatic vascular exclusion.

**SHVE: selective hepatic vascular exclusion.

↑: increase.

↓: reduction.

**Table 2:** Clinical series of hepatectomies performed under vascular occlusion techniques.

Technique-study	No. of patients	Type of hepatectomy^a^	Clamp time (min)	Morbidity/mortality (%)	Transfusions (%)	CVP (mmHg)
I.P^b^						
Torzilli et al. [36]	329	Major 71%	69	26/0	3.9	N/A
Nuzzo et al. [125]	120	Major 38%	39	N/A	60	<5
Omar Giovanardi et al. [126]	72	Major 81%	N/A	24/7	57	N/A
THVE^c^						
Smyrniotis et al. [54]	18	Major	32	33/0	30	N/A
Figueras et al. [124]	39	N/A	41	N/A	4	6.4
SHVE^d^						
Smyrniotis et al. [54]	20	Major	38	25/0	15	<5
Zhou et al. [58]	125	N/A	21.7	39.2/0	32	4.4
Fu et al. [127]	246	Major	N/A	24.8/0	24	2–5
Figueras et al. [124]	41	N/A	47	N/A	6	7.2
Pringle-IPM^e^						
Wang et al. [98]	114	N/A	N/A	N/A	13.1	5–10
Zhou et al. [58]	110	N/A	22.5	51.8/1.8	80.9	4.6
Ishizaki et al. [128]	380	Major 39.4%	62	23.9/0	34	N/A

^a^Major hepatectomy is defined as resection of more than two segments according to Couinaud's classification.

^b^I.P: ischemic preconditioning.

^c^THVE: total hepatic vascular exclusion.

^d^SHVE: selective hepatic vascular exclusion.

^e^IPM: intermittent pringle maneuver.
